# Keystone Veterinary Medical Association

**Published:** 1893-03

**Authors:** W. S. Kooker

**Affiliations:** Secretary


### SOCIETY PROCEEDINGS.

*Keystone Veterinary Medical Association.*—The regular meeting was held February 14th at the College of Physicians, President Dr. R. G. Webster in the chair. Drs. Hoskins, Kooker, Lintz, Webster, Lusson and John R. Hart answered roll-call. The minutes of previous meeting read, and, after the omission of Dr. Lintz's citing a case had been rectified, were approved. The essayists of the evening not being present, Dr. Hoskins addressed the members on meat and milk inspection, saying that the present law is so defective that it is almost an impossibility to get a conviction, if ever so guilty, consequently the Board of Health of this city propose to have amendments to strengthen the law and make it effective.

An Act to amend Section 1 of an Act entitled "An Act to Prevent the Adulteration of and Traffic in Impure and Unwholesome Milk in Cities of the second and third Classes," approved the 7th day of July, A.D. one thousand eight hundred and eighty-five, so as to make the said Act a general Act. It was moved and seconded that we indorse the said amendment. Passed.

An Act to amend Section 3 of an Act entitled "An Act to Prevent the Adulteration of and Traffic in Impure and Unwholesome Milk in Cities of the second and third Classes," approved the 7th day of July, A.D. one thousand eight hundred and eighty-five. This amendment to cover the whole State. Indorsed.

An Act to provide for licensing slaughter-houses in the several cities and boroughs of this commonwealth :

Section 1. Be it enacted by the Senate and House of Representatives of the Commonwealth of Pennsylvania, in General Assembly met, and it is hereby enacted by authority of the same, that from and after the first day of June next no slaughter-house shall be maintained in any city or borough of this commonwealth without a license therefor having been first obtained from the Board of Health of such city or borough, or, where no such board exists, from such other officers as the councils of such city or borough shall designate as the party to issue such license.

Section 2. The councils of the several cities and boroughs of this commonwealth shall provide, by ordinance, for the issuing of such license, and fix the fees therefor, which shall not, however, exceed ten dollars ($10) per annum.

Section 3. No person shall carry on any slaughter-house, in any city or borough, after such license-fee shall be fixed by ordinance, without first obtaining such license. Any person carrying on any slaughter-house without such license shall be guilty of a misdemeanor, and, upon conviction, shall be sentenced to pay a fine not exceeding one hundred dollars ($100), or undergo imprisonment not exceeding three months, or both, at the discretion of the Court.

Section 4. Any person carrying on a licensed slaughter-house shall, at all times, give free access to any inspector appointed by authority of law, either by the Agricultural Department of the United States Government, or by the State of Pennsylvania, or by the State Board of Health, or by the local Board of Health, or by the city or borough in which such slaughter-house is located.

Section 5. In any city or borough in which a license shall be provided for by ordinance, the Board of Health shall have authority to make rules and regulations governing such slaughter-houses; a violation of such rules and regulations to be punished by a revocation of the license, and by the imposition of such penalties as the said Board may deem proper, which penalties shall be pecuniary, and may be collected by suit, wherein the city or borough shall be plaintiff. Indorsed.

To amend an Act entitled "An Act to Prohibit the Sale of Unwholesome Meats," approved 7th day of May, 1855. The clause in the Act making it unlawful to kill and expose for sale veal less than three weeks old, is amended to read not less than five weeks old when killed, and weighing less than sixty pounds, dressed weight. Approved.

An Act to amend section 69 of an Act entitled "An Act to Consolidate, Revise and Amend the Penal Laws of this Commonwealth," approved the 31st day of March, 1860:

Section 1. Be it enacted by the Senate and House of Representatives of the Commonwealth of Pennsylvania, in General Assembly met, and it is hereby enacted by the authority of the same, that Section 69 of an Act entitled "An Act to Consolidate, Revise and Amend the Penal Laws of this Commonwealth," approved the 31st of March, 1860, which reads: "If any person shall sell or expose for sale the flesh of any diseased animal, or any other unwholesome flesh, knowing the same to be diseased or unwholesome, or sell or expose for sale unwholesome bread, drink or liquor, knowing the same to be unwholesome, or shall adulterate for the purpose of sale, or sell, any flour, meat or other article of food, any wine, beer, spirits of any kind, or other liquor intended for drinking, knowing the same to be adulterated, or shall adulterate for sale, or shall sell, knowing them to be so adulterated, any drugs or medicines, such person so offending shall be guilty of a misdemeanor, and, upon conviction," etc. Indorsed.

Dr. Hoskins next read the Act for the licensing of milk dealers and the inspection of dairies, etc. Approved.

W. S. KOOKER, *Secretary.*

